# Deep-learning model for predicting the survival of rectal adenocarcinoma patients based on a surveillance, epidemiology, and end results analysis

**DOI:** 10.1186/s12885-022-09217-9

**Published:** 2022-02-25

**Authors:** Haohui Yu, Tao Huang, Bin Feng, Jun Lyu

**Affiliations:** Department of Clinical Research, The First Affiliated Hospital of Jinan University, Guangzhou, 510630 China

**Keywords:** DeepSurv, Rectal adenocarcinoma, Neural network, Survival prediction, SEER

## Abstract

**Background:**

We collected information on patients with rectal adenocarcinoma in the United States from the Surveillance, Epidemiology, and EndResults (SEER) database. We used this information to establish a model that combined deep learning with a multilayer neural network (the DeepSurv model) for predicting the survival rate of patients with rectal adenocarcinoma.

**Methods:**

We collected patients with rectal adenocarcinoma in the United States and older than 20 yearswho had been added to the SEER database from 2004 to 2015. We divided these patients into training and test cohortsat a ratio of 7:3. The training cohort was used to develop a seven-layer neural network based on the analysis method established by Katzman and colleagues to construct a DeepSurv prediction model. We then used the C-index and calibration plots to evaluate the prediction performance of the DeepSurv model.

**Results:**

The 49,275 patients with rectal adenocarcinoma included in the study were randomly divided into the training cohort (70%, *n* = 34,492) and the test cohort (30%, *n* = 14,783). There were no statistically significant differences in clinical characteristics between the two cohorts (*p* > 0.05). We applied Cox proportional-hazards regression to the data in the training cohort, which showed that age, sex, marital status, tumor grade, surgery status, and chemotherapy status were significant factors influencing survival (*p* < 0.05). Using the training cohort to construct the DeepSurv model resulted in a C-index of the model of 0.824, while using the test cohort to verify the DeepSurv model yielded a C-index of 0.821. Thesevalues show that the prediction effect of the DeepSurv model for the test-cohort patients was highly consistent with the prediction resultsfor the training-cohort patients.

**Conclusion:**

The DeepSurv prediction model of the seven-layer neural network that we have established can accurately predict the survival rateand time of rectal adenocarcinoma patients.

## Background

Rectal cancer is a common malignant tumor of the digestive system [1] whose common histological types include adenosquamous carcinoma, adenocarcinoma, and undifferentiated carcinoma, with rectal adenocarcinoma accounting for more than 90% of cases [2]. Bray reported that there were approximately 700,000 new cases of rectal cancer and 310,000 deaths due to rectal cancer worldwide in 2018 [3]. Rectal cancer is the eighth-most-common type of cancer worldwide, and the ninth-most-common cause of death due to cancer. Rectal cancer mainly occurs in developed countries, withthose in North America ranking seventh in the world, wherethe incidence is 10.8 per 100,000 in males and 6.6 per 100,000 in females [3, 4]. A cancer report published by the American Cancer Society indicated that colorectal cancer was the third-most-common type of cancer in the United States in 2017, with its mortality rate ranked second among males and third among females [5]. Rectal cancer presents with atypical clinical symptoms in its early stages, which results in approximately 25% of patients already having metastases at the time of their first diagnosis [6, 7]. The 5-year survival rate is about 90% for early-stage rectal cancer,but less than 10% for advanced metastatic rectal cancer [7–9]. Developments in surgical techniques and the combined use of radiotherapy and chemotherapy in recent years have greatly improved the treatmentsapplied to patients with rectal cancer, but their mortality rate remains as high as 40% [10, 11]. Current treatment decisions and prognoses of rectal cancer patients are mainly based on the AJCC TNM staging system [8]. Different patients in the same stage of rectal cancer who receive similar treatmentscan exhibit large differences in treatment effects and survival rates [12]. Some studies have found that certain prognostic factors such as age, sex, and race might crucially affect survival predictions in individual patients [11–14].

Previous studies have used multiple types of assessment model to assess the survival rate of cancer patients, including the AJCC TNM staging system, logistics regression analysis, and the Cox proportional-hazards model [15–18]. The AJCC TNM staging system is currently the most commonly used tumor staging system worldwide [19], and it classifies cancer patients based on tumor and lymph node metastasis when evaluating and predicting their survival rate [20]. However, this method has disadvantages of a short evaluation time and data loss [21]. Logistics regression analysis identifies riskfactors that affect different outcomes [22]. However, this method has the disadvantage of losing temporal information that affects the ending event, which reduces its prediction ability [23]. The Cox proportional-hazards model includes survival outcomes and survival time as dependent variables. This model can be used to simultaneously analyze the impact of multiple factors on survival time, and it is widely used to predict outcome events without knowledge of the survival distribution of the analyzed data [24, 25]. A nomogram is a widely used method for combining and quantifying various important clinical characteristics of patients when calculating the probabilities of outcome events occurring based on Cox proportional-hazards model [26]. However, an assumption underlying the Cox proportional-hazards model is that each predictor variable has the same impact at the follow-up time, which ignores differences in the impact of predictor variables on individual patientsat different times [24]. Therefore, a new method is neededthat has a higher accuracy in predicting the survival rate of cancer patients.

Developmentsincomputer and information technology over recent years havemade it possible to improve the accuracy of predictions of the survival rate of cancer patients [27]. Deep learning is a new research direction in the field of machine learning that involves discovering the distributed characteristics of sample data by learning the underlying laws and representation levels [28]. Deep learning is essentially a statistical model that includesan input layer, hidden layer, and output layer, which can be used to solve multifactor and nonlinear problems. The continuous developmentsin deep-learning research methods and the availability of biomedical big data have led to machine learning being used to predict the clinical outcomes of patients [29]. Liu et al. reported that an artificial neural-network model can be applied to clinical information to predict the survival rate of patients with nasopharyngeal cancer [30]. Katzman et al. combined deep learning with a multilayer neural network (the DeepSurv model) to develop a system for personalized treatment recommendations [31]. The present study collected data on patients with rectal adenocarcinoma in the United States from the Surveillance, Epidemiology, and End Results (SEER) database and applied the DeepSurv model to investigate their survival rates.

## Method

### Data source

All of the patients with rectal adenocarcinoma included in this study were selected from the SEER “18 Regs Custom Data Nov 2017 Sub (1973-2015 varying)” data set with additional treatment fields (http://seer.cancer.gov). The SEER database contains data on cancer patients from 18 regions of the United States, and accounts for around 28% of the total country population [32]. This database contains a considerable amount of relevant information on patients, including demographic data, tumor data, and information on causes of death and survival times. We used SEER*Stat software (version 8.3.6) to identify patients in the data set who had rectal adenocarcinoma in the United States from 2004 to 2015. We obtained permission to access the database by signing the SEER Research Data Agreement form and submitting it via email.

### Inclusion and exclusion criteria for the study population

**Fig. 1:**
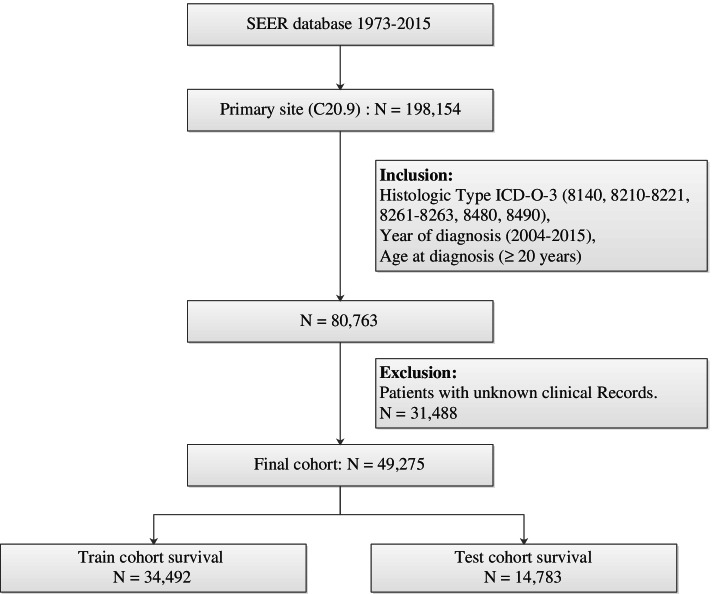
The flow diagram of patients with rectal adenocarcinoma selection

We identifiedpatients with rectal adenocarcinoma using primary site code C20.9 of the third revision of the International Classification of Diseases for Oncology codes (ICD-O-3) along with rectal and morphology codes 8140, 8210–8221, 8261–8263, 8480, and 8490. The inclusioncriteria for the study population includedbeing diagnosedduring 2004–2015 and aged> 20 years, while theexclusion criteria included the first tumornot being rectal adenocarcinoma and unknown tumor grade, survival time, race, marital status, or surgery status. We screened 49,275 patients with rectal adenocarcinoma and collected the following information from the SEER database:sex, age, marital status, race, tumor grade, AJCC TNM stage, tumor size, tumor location, degree of tumor invasion, surgery status, radiotherapy status, chemotherapy status, survival time, and cause of death. We divided the collected rectal adenocarcinoma patients into the following four groups based on ICD-O-3 morphology codes: papillary adenocarcinoma (code 8140), tubular adenocarcinoma (codes 8210–8221 and 8261–8263), mucinous adenocarcinoma (code 8480), and signet-ring-cell carcinoma (code 8490). We recoded marital status into married and unmarried, where the latter status included single, unmarried, widowed, separated, and divorced. We subsequently randomly divided the patients into training and test cohortsat a ratio of 7:3. Figure 1 shows the screening procedure applied to identify patients with rectal adenocarcinoma.

### Design and analysis of deep-learning models

**Fig. 2:**
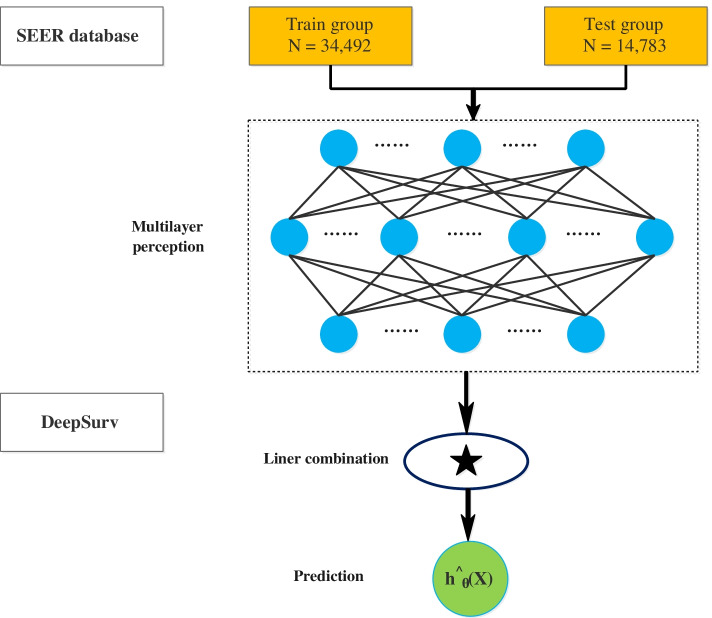
Diagram of the deep learning procedure

DeepSurv is a deep feedforward neural network that can be used to predict the effects of patient covariates on patient survival. The structure of this network includeshuge numbers of simulated neurons that are divided into three main layers: input, hidden, and output layers. There can only be one input layer and one output layer, while there can be multiple hidden layers (Fig. 2). We performed deep-learning calculations based on the DeepSurv calculation method described by Katzman et al. [31] to predict the survival outcome of patients with rectal adenocarcinoma. The training-cohort data were used to develop a DeepSurv model of a seven-layer neural network. We then used the test-cohort data to perform DeepSurv analysis to evaluate the effectiveness of the model and predict the survival rate of patients with rectal adenocarcinoma. Finally, we used Harrell C statistics and correction graphs to evaluate the prediction performance in the training and test cohorts.

### Statistical analysis

Python software (version 3.7.6) was used to perform all computations and analyses in thisstudy. We first used the Pandas library to perform a basic statistical analysis of the data. Kaplan-Meier analysis and log-ranktesting were then performed using the Python lifelines survival analysis module. Meanwhile, sklearn was used to randomize the data and normalize the mean and variance. A *k*-fold check (*k* = 10) was used in the model training process to ensure its accuracy. We finally used Python combined with the deep-learning framework theano to complete the simulations. All tests were double-sided, and the significance criterion was set to *p* < 0.05.

## Results

### Baseline characteristics of the patients

The 49,275 included patients with rectal adenocarcinoma comprised 29,504 male patients (59.9%) and 19,771 female patients (40.1%). The basic clinical characteristics in the two study cohorts are listed in Table 1, which indicates that none of the clinical characteristics differed significantly between the cohorts (*p* > 0.05). The patients were aged 62.6 ± 13.5 years (mean ± SD), and most of them were white (81.3%), had grade II tumors (76.2%), and papillary adenocarcinoma (74.2%). The maximum follow-up time for patients was 143 months, with a mean of 47 months. During the study period from 2004 to 2015, 14,078 (28.5%) patients died of rectal adenocarcinoma.

**Table 1:** Analysis of the main characteristics of patients with rectal adenocarcinoma

Variables	Overall N(%)	Train cohort N(%)	Test cohort N(%)	***P***
Patients	49,275	34,492(70.0%)	14,783(30.0%)	
Sex				
Female	19,771(40.1%)	13,878(40.2%)	5893(39.9%)	0.440
Male	29,504(59.9%)	20,614(59.8%)	8890(60.1)	
Age at diagnosis				
mean ± sd	62.6 ± 13.5	62.5 ± 13.5	62.7 ± 13.4	0.131
Race				
Black	4174(8.5%)	2951(8.6%)	1223(8.3%)	0.326
White	40,063(81.3%)	28,053(81.3%)	12,010(81.2%)	
Amercian Indian/Alaska Native	390(0.8%)	279(0.8%)	111(0.8%)	
Asian or Pacific Islander	4648(9.4%)	3209(9.3%)	1439(9.7%)	
Marital status at diagnosis				
Married	29,160(59.2%)	20,482(59.4%)	8678(58.7%)	0.160
Unmarried	20,115(40.8%)	14,010(40.6%)	6105(41.3%)	
Histologic^**#**^				
PA	36,549(74.2%)	25,564(74.1%)	10,985(74.3%)	0.847
TA	10,364(21.0%)	7285(21.1%)	3079(20.8%)	
MA	1955(4.0%)	3143(3.9%)	598(4.0%)	
SRCC	407(0.8%)	423(0.9%)	121(0.9%)	
Grade				
Grade I	4537(9.2%)	3143(9.1%)	1394(9.4%)	0.717
Grade II	37,522(76.2%)	26,298(76.3%)	11,224(76.0%)	
Grade III	6616(13.4%)	4628(13.4%)	1988(13.4%)	
Grade IV	600(1.2%)	423(1.2%)	177(1.2%)	
AJCC				
I	15,009(30.5%)	10,501(30.5%)	4508(30.5%)	0.888
II	10,899(22.1%)	7601(22.0%)	3298(22.3%)	
III	15,048(30.5%)	10,546(30.6%)	4502(30.5%)	
IV	8319(16.9%)	5844(16.9%)	2475(16.7%)	
T				
T0	7(0.01%)	5(0.01%)	2(0.01%)	0.616
T1	10,071(20.4%)	7071(20.5%)	3000(20.3%)	
T2	7940(16.1%)	5502(16.0%)	2438(16.5%)	
T3	23,928(48.6%)	16,786(48.7%)	7142(48.3%)	
T4	4089(8.3%)	2832(8.2%)	1257(8.5%)	
TX	2111(4.3%)	1494(4.3%)	617(4.2%)	
Tis	1123(2.3%)	796(2.3%)	327(2.2%)	
N				
N0	28,582(58.0%)	19,965(57.9%)	8617(58.3%)	0.765
N1	13,613(27.6%)	9577(27.8%)	4036(27.3%)	
N2	5936(12.1%)	4148(12.0%)	1788(12.1%)	
NX	1144(2.3%)	802(2.3%)	342(2.3%)	
M	0			
M0	40,934(83.1%)	28,633(83.0%)	12,301(83.2%)	0.848
M1	8319(16.9%)	5844(16.9%)	2475(16.7%)	
MX	22(0.05%)	15(0.1%)	7(0.1%)	
Summary stage	0			
Regional	20,563(41.7%)	14,358(41.6%)	6205(42.0%)	0.755
Distant	19,956(40.5%)	13,986(40.5%)	5970(40.4%)	
Localized	8756(17.8%)	6148(17.9%)	2608(17.6%)	
Surgery performed	0			
No	8367(17.0%)	5877(17.0%)	2490(16.8%)	0.597
Yes	40,908(83.0%)	28,615(83.0%)	12,293(83.2%)	
Radiotherapy	0			
No/Unknown	21,408(43.4%)	15,019(43.5%)	6389(43.2%)	0.505
Yes	27,867(56.6%)	19,473(56.5%)	8394(56.8%)	
Chemotherapy	0			
No/Unknown	18,286(37.1%)	12,836(37.2%)	5450(36.9%)	0.464
Yes	30,989(63.1%)	21,656(62.8%)	9333(63.1%)	
Status	0			
Death	14,078(28.5%)	9871(28.5%)	4207(28.5%)	0.790
Alive	35,247(71.5%)	24,671(71.5%)	10,576(71.5%)	

^#^*PA* Papillary adenocarcinoma, morphology code 8140; *TA* Tubular adenocarcinoma, morphology code 8210–8221, 8261–8263; *MA* Mucinous adenocarcinomas, morphology code 8480; *SRCC* Signet ring cell carcinoma, morphology code 8490

### Coxproportional-hazards regression and DeepSurv curve in the training cohort

**Fig. 3:**
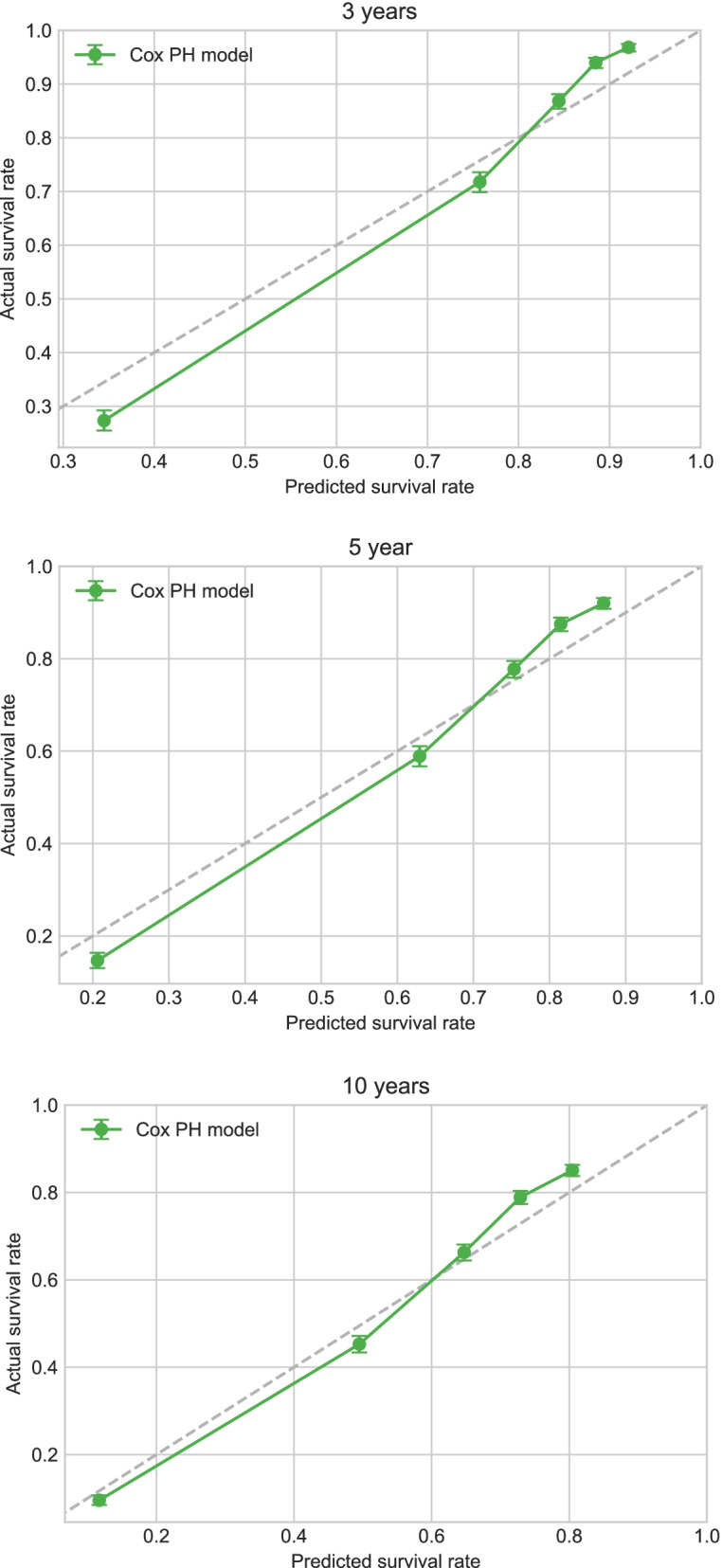
Calibration plots of survival rate of rectal adenocarcinoma in Cox PH model

Applying Cox proportional-hazards regression to the data in the training cohort showed that age, sex, marital status, tumor grade, surgery status, and chemotherapy status significantly affected their survival (*p* < 0.05) (Table 2). The C-index for the Cox proportional-hazards regression model was 0.788. We produced calibration charts of the Cox proportional-hazards model for the 3-, 5-, and 10-year survival of rectal adenocarcinoma patients in the training cohort,which revealed some discrepancies between the predictions of the Cox proportional-hazards regression model and the actual events (Fig. 3).

**Table 2:** Survival predictors in Cox PH model

Variables	β	HR	95%CI	***P***
Age at diagnosis	0.02	1.02	1.01–1.03	< 0.005**
Race	0.06	1.07	1.04–1.09	< 0.005**
Sex	−0.07	0.93	0.90–0.97	< 0.005**
Marital status	−0.26	0.77	0.75–0.80	< 0.005**
Histologic	−0.04	0.96	0.94–0.99	0.02*
Grade	−0.09	0.91	0.90–0.92	< 0.005**
AJCC stage	−0.02	0.98	0.96–1.00	0.05*
T stage	0.06	1.06	1.05–1.07	< 0.005**
N stage	0.20	1.22	1.20–1.24	< 0.005**
M stage	0.60	1.83	1.70–1.96	< 0.005**
Summary_stage	0.06	1.06	1.02–1.09	< 0.005**
Surg Prim Site^a^	0.00	1.00	1.00–1.00	0.01*
Surgery	−0.83	0.43	0.41–0.46	< 0.005**
Chemotherapy	−0.21	0.81	0.77–0.84	< 0.005**
CS tumor size (2004+)^b^	0.00	1.00	1.00–1.00	< 0.005**
CS extension (2004+)^c^	0.00	1.00	1.00–1.00	< 0.005**
CS_lymph_nodes (2004+)^d^	0.00	1.00	1.00–1.00	0.04*
CS mets at dx (2004+)^e^	0.01	1.01	1.01–1.02	< 0.005**

*Cox PH* Cox proportional hazard regression; *HR* Hazard Ratio; *CI* Confidence Interval

^a^Surg Prim Site:Surgery of Primary Site describes a surgical procedure that removes and/or destroys tissue of the primary site performed as part of the initial work-up or first course of therapy

^b^CS tumor size (2004+): Information on tumor size. Available for after 2004 year. Earlier cases may be converted and new codes added which weren’t available for use prior to the current version of CS

^c^CS extension (2004+):Information on extension of the tumor. Available for after 2004 year. Earlier cases may be converted and new codes added which weren’t available for use prior to the current version of CS

^d^CS_lymph_nodes (2004+): Information on involvement of lymph nodes. Available for after 2004 year. Earlier cases may be converted and new codes added which weren’t available for use prior to the current version of CS

^e^CS mets at dx (2004+): Information on distant metastasis. Available for after 2004 year. Earlier cases may be converted and new codes added which weren’t available for use prior to the current version of CS

* *P* < 0.05, ** *P* < 0.01, *** *P* < 0.001

**Fig. 4:**
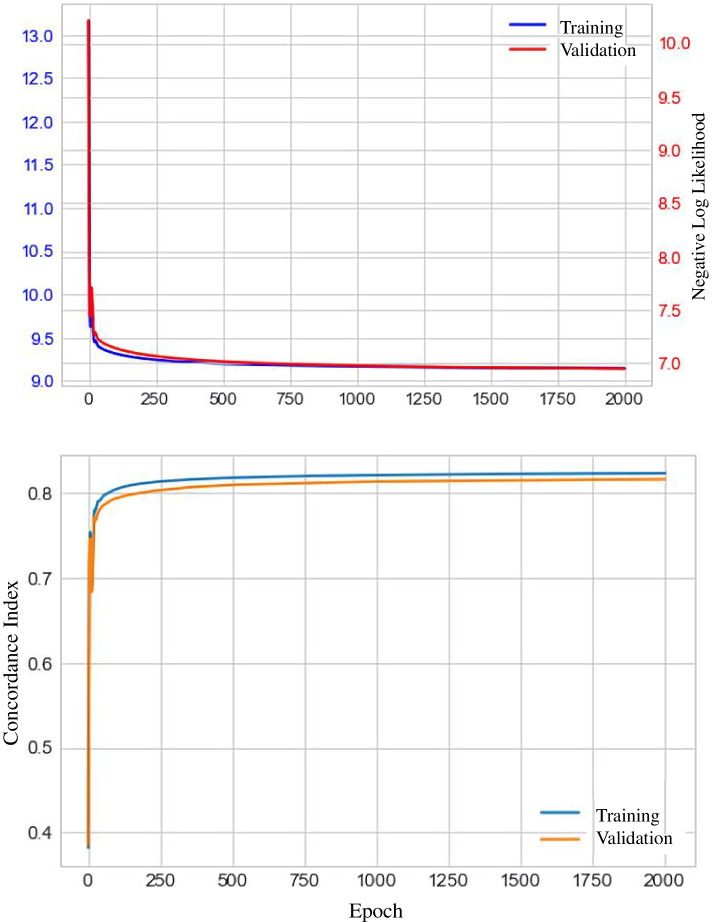
The plots of the training cohort C index and loss function

**Fig. 5:**
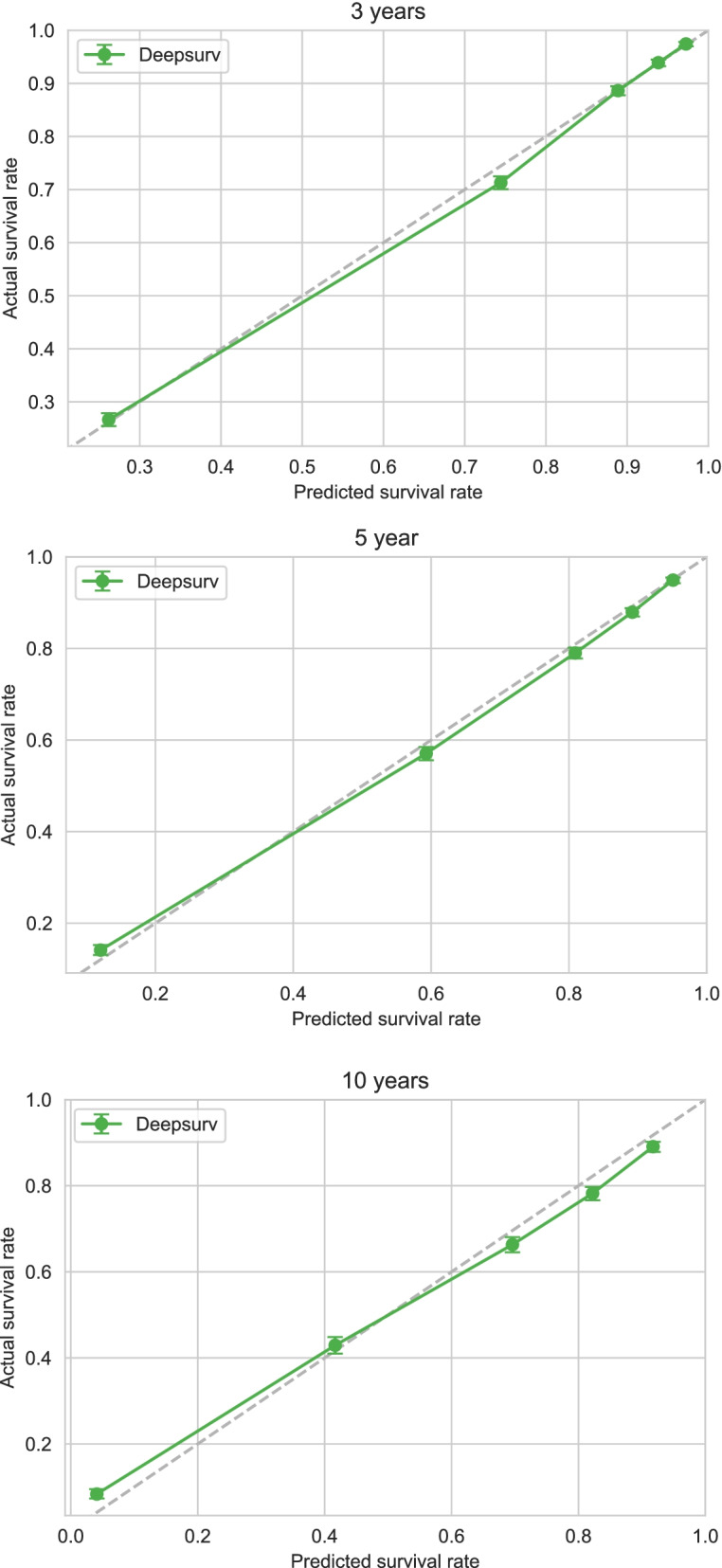
Calibration plots of the survival rate of the training cohort in the DeepSurv model

The C-index obtained when using the training-cohort data to construct the DeepSurv model was 0.824. The graph of the training-cohort C-index and loss function is shown in Fig. 4. The calibration chart of the DeepSurv model for the survival of training-cohort patients at 3, 5, and 10 years also revealed discrepancies between the predictions of the DeepSurv model and the actual events (Fig. 5). However, the predictions of the DeepSurv model were better than those based on the Cox proportional-hazards regression model.

### Calibration and verification of the DeepSurv model in the test cohort

**Fig. 6:**
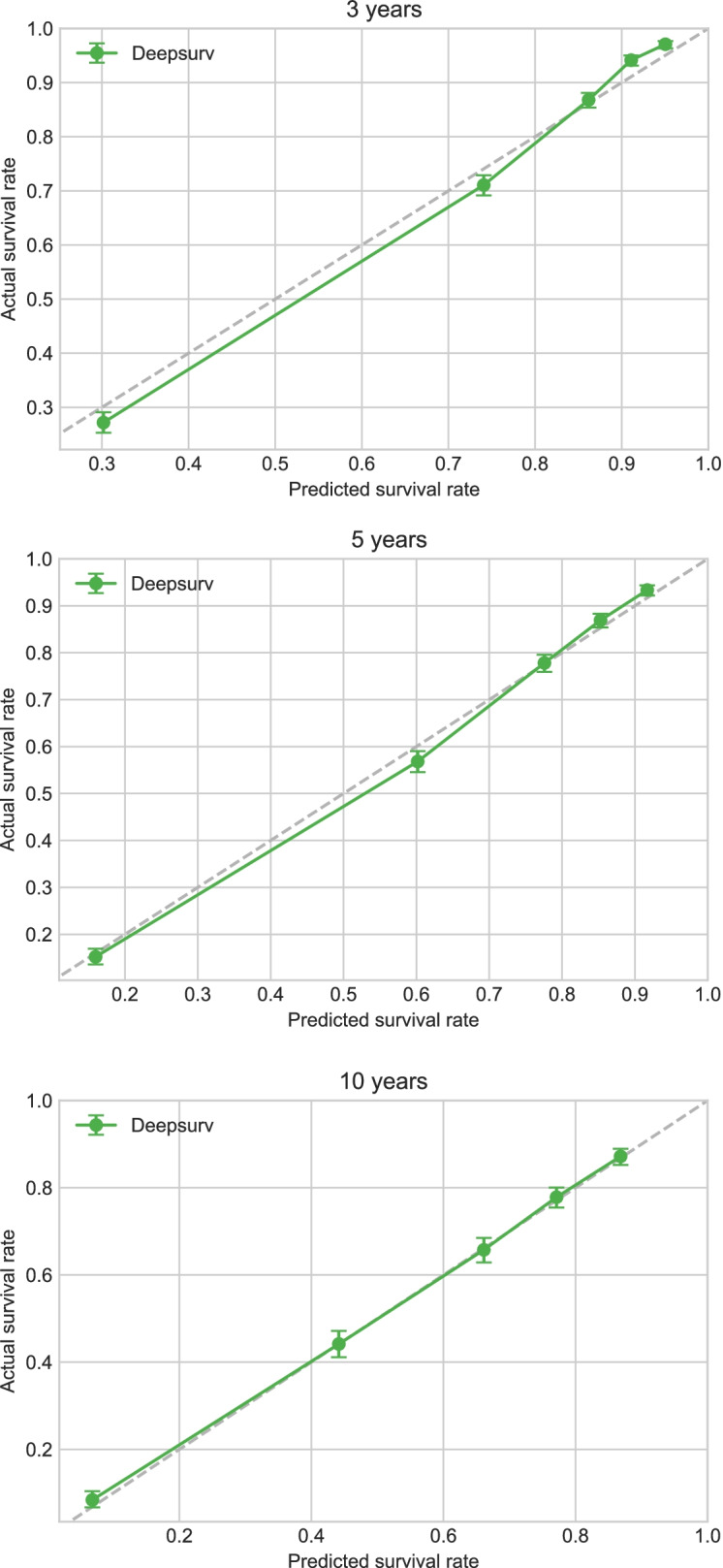
Calibration plots of the survival rate of the test cohort in the DeepSurv model

Applying the variables selected by the Cox proportional-hazards regression model of the training cohort to the test cohortwith the DeepSurv model showed that the latter had a good predictive effect, with a C-index of 0.821. The calibration curves for the survival of patients in the test cohort at 3, 5, and 10 years are presented in Fig. 6, which shows that the predictions of the DeepSurv model for the test-cohort patients are highly consistent with the prediction results for the training-cohort patients.

### Comparison between the DeepSurv model and the AJCC TNM staging system

**Fig. 7:**
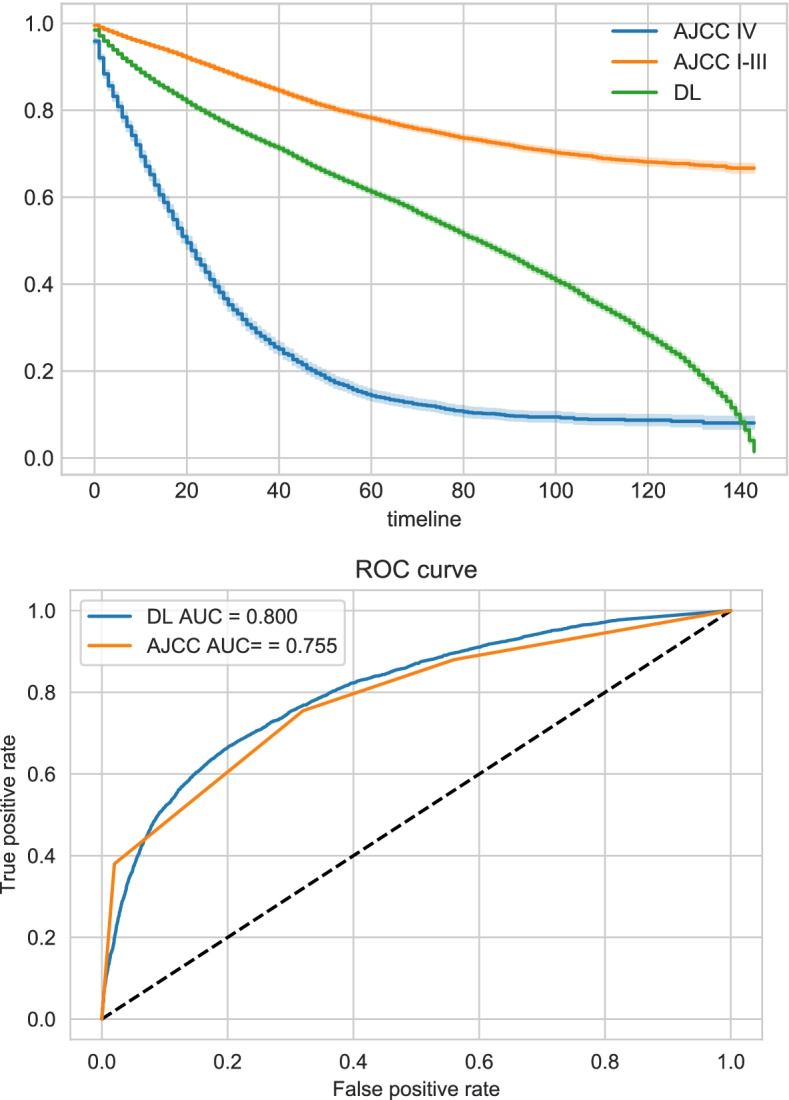
Comparison of survival curve and AUC between DeepSurv model and AJCC stage system

The AJCC TNM stages were dichotomized into stages I–III and stage IV based on the presence of distant metastasis, which corresponded to no distant transfer and distant transfer, respectively. Figure 7 shows that the survival rate was significantly lower for patients at stages I–III than for those at stage IV. That figure shows that the DeepSurv model predicted that the survival risk was lower than for patients classified as AJCC TNM stages I–III, and higher than for those classified as AJCC TNM stage IV. Moreover, the survival curve was smoother for the DeepSurv model than for the AJCC TNM staging system. The area under the receiver operating characteristic (ROC) curve (AUC) was larger for the DeepSurv model than for the AJCC TNM staging system, while the latter ROC curve was located above and to the left of that for the AJCC TNM staging system. The results showed that the DeepSurv model was more accurate in predicting the survival prognosis of rectal adenocarcinoma patients compared with the AJCC TNM staging system.

## Discussion

Rectal adenocarcinoma is a common clinical malignant tumor that is reasonably common in developed countries, including those in North America and Europe [3, 4]. Tumor metastasis is reportedly present in more than 50% of newly diagnosed patients, which is due to the atypical clinical symptoms of early-stage rectal adenocarcinoma [7]. Effective methods for the early detection and early treatment of rectal adenocarcinoma would therefore be of great significance for improving the prognosis of affected patients. Various risk factors affecting the prognosis of these patients have been reported in recent years, including age, sex, histological type, tumor stage, and tumor differentiation status [33, 34].

With the aim of improving the accuracy of survival-time predictions for patients with rectal adenocarcinoma, various methods have been used to establish prediction models, including the AJCC TNM staging system, logistics regression analysis, and the Cox proportional-hazards model [15–18]. Each of these prediction models has certain advantages and disadvantages, and different models produce different predictions of patient survival. The Cox proportional-hazards model is currently one of the most widely used models for prognostic predictions [26], and such models require each predictor variable to be a linear factor, which therefore ignores the impacts of any significant nonlinear factors on outcome variables. It is well known that the development of tumors and changes therein are affected by many factors, and so traditional linear models are highly unlikely to accurately predict the prognosis of cancer patients. This situation makes it necessary to develop new methods that can combine linear and nonlinear factors in the construction of prediction models.

The ongoing developments in computer and information technology can facilitate the construction of the required novel predictive models. For example, Katzman et al. implemented the DeepSurv analysis method by combining deep learning with a multilayer neural network [31]. The DeepSurv method includes a complex three-layer network structure comprising input, hidden, and output layers [29]. The input layer includes each linear or nonlinear predictor variable, the hidden layer has a multilayer structure for variable conversion, and the output layer is the converted target variable. The DeepSurv method uses deep-learning technology to convert multiple linear and nonlinear factors into a linear combination via multilevel fusion and transformation to predict outcome events. The DeepSurv approach is being gradually applied in various fields related to biomedical research. Multiple research results have shown that the predictions made using the DeepSurv model are better than those made using traditional linear prediction models [35–37]. She et al.used a DeepSurv model to provide non-small-cell lung-cancer-specific survival and prognosis predictions as well as treatment recommendations, and found that its prediction effect was significantly better than that of the traditional AJCC TNM staging system [38]. Biglarian et al. demonstrated that the DeepSurv model is superior to the Cox proportional-hazards model in predicting distant metastasis in patients with rectal cancer [39]. Rau et al. found that a DeepSurv model for predictions associated with liver cancer was superior to those obtained using a logistic regression model [40].

This study constructed a DeepSurv model of the survival rate of rectal adenocarcinoma patients by collecting affected patients living in the United States from the SEER database. We first conducted a Cox proportional-hazards regression analysis of 34,492 patients with rectal adenocarcinoma in the training cohort to identify risk factors for their prognosis. These risk factors were age, race, sex, marital status, tumor grade, AJCC TNM stage, surgery status, chemotherapy status, tumor size, and degree of tumor invasion (*p* < 0.05) (Table 1). We then developed a seven-layer neural-network DeepSurv prediction model based on the analytical method established by Katzman et al. [31] The C-index when applying the new prediction model was 0.821 for the test cohort and 0.824 for the training cohort. These values show that the predictions of the DeepSurv model for the test-cohort patients are highly consistent with those for the training-cohort patients. The results obtained for the calibration curves of the patients in the test cohort at 3, 5, and 10 years further support this conclusion. The DeepSurv model was also found to provide more accurate predictions of the prognosis of patients with rectal adenocarcinoma compared with the Cox proportional-hazards model, which is consistent with the results of some previous studies of cancer prognoses. It has also been shown previously that the DeepSurv model provides powerful variable-processing capabilities [35, 41]. Finally, we compared the DeepSurv prediction model with the AJCC TNM staging system, and found that the AUC was higher for the former (AUC = 0.800) than the latter (AUC = 0.755). Meanwhile, the survival curve was smoother for the DeepSurv model than for the AJCC TNM staging system. The superior results for the survival prognosis of patients with rectal adenocarcinoma obtained by applying the DeepSurv model are due to it transforming linear and nonlinear predictive variables into a linear combination by utilizing a multilevel neural network [31]. Deep learning can be used to solve nonlinear problems involving multiple factors, and so the DeepSurv model has particular advantages over other models when dealing with large samples, multiple variables, and nonlinearity.

The present study was subject to some limitations. First, some potentially information that might affect survival was missing for the patients with rectal adenocarcinoma collected from the SEER database, such as whether tumors were surgically removed, the type of chemotherapy applied, medications, the psychological status, religious beliefs, and education of the patients, and their familial tumor history. Second, our study only included data for patients with rectal adenocarcinoma living in certain parts of the United States, and the established DeepSurv prediction model was not validated using external data. The accuracy of the DeepSurv approach could be further assessed using patients with rectal adenocarcinoma living in other countries. Third, the DeepSurv model has its own inherent limitations during the construction process. The existence of hidden layers in the black-box model meant that we cannot exactly understand the calculations performed during the model construction process, or the associated limitations. Future studies should attempt needed to resolve the above-mentioned problems.

## Conclusions

This study used Cox proportional-hazards regression analysis to identify the risk factors affecting the prognosis of rectal adenocarcinoma patients, which include age, sex, tumor grade, tumor size, degree of tumor invasion, surgery status, and chemotherapy status. We constructed a seven-layer neural-network DeepSurv prediction model that has been demonstrated to provide good predictions of the prognosis of patients with rectal adenocarcinoma. This novel DeepSurv model can be used to accurately predict the survival time of patients with rectal adenocarcinoma.

## Data Availability

We obtained permission to access the database after signing and submitting the SEER Research Data Agreement form via email. The data that support the findings of this study are available from SEER database but restrictions apply to the availability of these data, which were used under license for the current study, and so are not publicly available. Data are however available from the authors upon reasonable request and with permission of SEER database.
